# Supplementary material for: Risk Factors for the Mental Health of Adolescents from the Parental Perspective: Photo-Voice in Rural Communities of Ecuador
Source: Int J Environ Res Public Health. 2023 Jan 26;20(3):2205. doi: 10.3390/ijerph20032205 (PMC9915978; doi:10.3390/ijerph20032205)
Supplement: Supplementary file 1 [file ijerph-20-02205-s001.zip › ijerph-2104946-supplementary.pdf]

Appendix

Transformation of knowledge

Deductive Category Assignments	Definition	Anchor examples
Acquisition or modification of information	Parents can identify the acquire knowledge or preconceived notions about mental health risk factors.	"This is new…"; "I did not know that…"; "I believed that…"
Problem analysis	Parents can identify problems that make adolescents more likely to will develop a mental health affectation in different dimensions of the daily life.	"Lack of…"; "May be that…"; "They look…"
Solving activities	Parents communicate their opinions, concerns, and possible solution to approach risk factors for the mental health of adolescents.	"Experiential approaches with photo-voice"; Community-based participatory research"; "Abogacy"

Based on theoretical review and qualitative contents analysis method.
